# LDL-MobileNetV3S: an enhanced lightweight MobileNetV3-small model for potato leaf disease diagnosis through multi-module fusion

**DOI:** 10.3389/fpls.2025.1656731

**Published:** 2025-10-22

**Authors:** Jinyan Zhang, Xiaofei Yang, Xueliang Fu, Buyu Wang, Honghui Li

**Affiliations:** ^1^ College of Computer and Information Engineering, Inner Mongolia Agricultural University, Hohhot, China; ^2^ Key Laboratory of Smart Animal Husbandry at Universities of Inner Mongolia Autonomous Region, Hohhot, China

**Keywords:** potato leaf disease, MobileNetV3 Small, Lite Fusion, Dynamic Dilated Convolution, Lightweight Attention

## Abstract

**Introduction:**

The timely and precise detection of foliar diseases in potatoes, a food crop of worldwide importance, is essential to safeguarding agricultural output. In complex field environments, traditional recognition methods encounter significant challenges, including the difficulty in extracting features from small and diverse early-stage lesions, blurred edge features due to gradual transitions between diseased and healthy tissues, and degraded robustness from background interference such as leaf texture and varying illumination.

**Methods:**

To address these limitations, this study proposes an optimized lightweight convolutional neural network architecture, termed LDL-MobileNetV3S. The model is built upon the MobileNetV3 Small backbone and incorporates three innovative modules: a Lightweight Multi-scale Lite Fusion (LF) module to enhance the perception of small lesions through cross-layer connections, a Dynamic Dilated Convolution (DDC) module that employs deformable convolutions to adaptively capture pathological features with blurred boundaries, and a Lightweight Attention (LA) module designed to suppress background interference by assigning spatially adaptive weights.

**Results:**

Experimental results demonstrate that the proposed model achieves a recognition accuracy of 94.89%, with corresponding Precision, Recall, and F1-score values of 93.54%, 92.53%, and 92.77%, respectively. Notably, these results are attained under a highly compact model configuration, requiring only 6.17 MB of storage and comprising 1.50 million parameters. This is substantially smaller than benchmark models such as EfficientNet-B0 (15.61 MB / 3.83 M parameters) and ConvNeXt Tiny (106 MB / 27.8 M parameters).

**Conclusion:**

The proposed LDL-MobileNetV3S model demonstrates superior performance and efficiency compared to several existing lightweight models. This study provides a cost-effective and high-accuracy solution for potato leaf disease diagnosis, which is particularly suitable for deployment on intelligent diagnostic devices operating in resource-limited field environments.

## 1 Introduction

As a widely cultivated staple crop worldwide, potato holds substantial nutritional and economic value, playing a critical role in safeguarding food security and promoting the growth of cash crop industries (Kharumnuid et al., 2021). Effective management of potato production is crucial for ensuring both food supply stability and the profitability of agricultural systems. However, throughout the growth cycle of potatoes, their leaves are frequently affected by various diseases, including late blight, early blight, and viral infections (Tejas et al., 2023). These diseases compromise plant health, reduce yield, and degrade product quality, ultimately causing significant economic losses in agriculture. Therefore, achieving efficient and accurate detection of potato leaf diseases is crucial for effective disease management, intelligent agricultural practices, and the improvement of crop productivity (Kaur et al., 2024).

Traditional methods for identifying plant diseases primarily rely on manual field observation and the subjective judgment of agricultural specialists. While these approaches may yield acceptable accuracy in localized scenarios, they often suffer from low efficiency, inconsistent results, and limited scalability. Moreover, they are inadequate for meeting the demands of modern precision agriculture, which requires real-time, data-driven decision-making across large and diverse field conditions. Consequently, traditional techniques fall short in supporting high-throughput, automated monitoring essential for large-scale crop management (Khakimov et al., 2022) (Liu and Wang, 2021).

In recent decades, machine vision and artificial intelligence have developed rapidly. Image recognition techniques driven by deep learning have found widespread use in diagnosing plant diseases (Bhargava et al., 2024). Convolutional Neural Networks (CNNs) have become a focus of research. They can automatically learn image features with strong efficiency. CNNs have demonstrated strong capabilities in identifying leaf diseases and locating affected regions (Lu et al., 2021). CNNs are capable of extracting critical features such as color, texture, edge, and structural information from images. This is achieved through a series of multi-layer nonlinear transformations. Such processing reduces dependence on traditional handcrafted feature design. It also enhances the automation and generalization capabilities of plant disease recognition systems. These technologies offer a promising pathway for deploying low-cost, automated monitoring systems in agricultural fields, greenhouses, and rural environments. For example, Atila et al. (2021) conducted a systematic evaluation using the PlantVillage dataset, which includes 54,306 images. Under a five-fold cross-validation strategy, the EfficientNet-B4 and ResNet50 architectures achieved average classification accuracies exceeding 99%. These results significantly surpassed those of traditional machine learning approaches. Sutaji and Yıldız (2022) proposed a lightweight feature extraction model based on the MobileNetV2 and Xception architectures. The model incorporated a multi-scale depthwise separable convolution structure to improve recognition accuracy. It maintains a low parameter count and computational cost. These characteristics make the model adaptable to mobile agricultural platforms, such as handheld diagnostic devices or drone-mounted systems. Liu et al. (2022a) proposed a hybrid deep learning framework named DenseACNet. The model integrated a channel attention mechanism with data augmentation strategies to enhance the accuracy and robustness of crop disease recognition. This approach achieved strong classification performance on the extended PlantVillage dataset. These studies demonstrate that CNN-based approaches hold strong potential for application in agricultural image analysis.

However, despite their strong recognition performance, deep CNN models still face major limitations. Their large parameter sizes and high computational costs hinder deployment on edge devices, unmanned aerial platforms, and mobile smart farming systems (Abade et al., 2021) (Zawish et al., 2024). For instance, He et al. (2016) introduced the well-known ResNet architecture and developed a deep ResNet-101 model. This model contained over 44 million parameters and requires approximately 7.6 GFLOPs for inference. While it delivered strong results on high-performance servers, it posed major challenges for deployment in resource-constrained environments. Simonyan and Zisserman et al. (Simonyan and Zisserman, 2014) developed the classical VGG-16 model, which achieved high classification accuracy on the ImageNet dataset. However, the model contains 138 million parameters and requires over 15 GFLOPs for inference. These characteristics limit its ability to meet the dual demands of real-time performance and energy efficiency in edge computing environments. To address this issue, researchers have proposed various lightweight network architectures, including the MobileNet family (Howard et al., 2017) (Sandler et al., 2018) (Howard et al., 2019), EfficientNet (Tan and Le, 2019), and ShuffleNet (Zhang et al., 2018). These models reduce parameter size and computational cost by employing techniques such as depthwise separable convolution, neural architecture search, and channel pruning. Such methods enhance deployment efficiency while maintaining recognition accuracy. In the context of smart agriculture, these lightweight models provide a foundation for scalable, real-time monitoring systems applicable to diverse field conditions.

Although lightweight networks offer advantages for deployment, they still encounter major challenges. These include early-stage disease detection, complex background interference, and the identification of small lesion areas (Mohanty et al., 2016). To address these issues, recent studies have increasingly integrated structural optimization with modular enhancements. This approach aims to improve the semantic representation capacity of lightweight models. Specific methods include the Attention Mechanism (Woo et al., 2018), Dilated Convolution (Chen et al., 2018), and Multi-scale Feature Fusion (Lin et al., 2017) (Liu et al., 2018). Woo et al. (2018) introduced the CBAM (Convolutional Block Attention Module), which combines channel and spatial attention mechanisms. This design enhances the model’s discriminative capability in image recognition tasks. Many plant disease studies apply CBAM to focus on key lesion regions and enhance saliency modeling. Xu et al. (2022) developed a multi-scale dilated convolution structure to achieve sparse receptive field coverage. This design strengthened the model’s ability to identify blurred leaf edges and irregularly shaped lesions. Ren et al. (2023) used a multi-layer feature fusion strategy to build shallow enhancement paths. This approach increased the sensitivity to small lesion areas. These modular integration strategies help improve the semantic representation capability of lightweight networks. Nevertheless, achieving a balance between model accuracy and computational efficiency remains a key challenge in practical deployment. For example, the plant disease classification model proposed by Sholihati et al. (2020), which is based on the VGG-16 architecture, demonstrated high recognition accuracy. However, due to its substantial parameter count (138 million) and significant computational cost (15.3 billion FLOPs), the model faces limitations in adapting to the constrained resources of edge computing environments. Charisma and Adhinata (2023) applied the DenseNet201 model, which showed strong performance in extracting features from plant leaves. However, its high computational complexity limited its suitability for real-time detection tasks. Likewise, Khan et al. (2020) introduced a tomato disease identification method based on ResNet50 combined with saliency graph analysis. Although the model achieved 98.6% accuracy on the PlantVillage dataset, its computational load (23 million parameters and 4 billion FLOPs) limited its applicability in edge environments. These studies indicate that enhancing the feature extraction capability of lightweight models for small target detection remains a central challenge in plant disease recognition. Optimizing such models is essential for balancing detection accuracy and computational efficiency. This challenge is particularly critical in agricultural settings, where timely and efficient on-site analysis is vital for early disease intervention and minimizing crop losses.

Based on these observations, this study introduces a lightweight neural network that integrates multiple modules and builds upon the MobileNetV3 Small architecture. The model aims to achieve high-precision recognition of potato leaf lesions while maintaining suitability for deployment on low-power devices. The main contributions of this work are reflected in the following three innovations: (1) The proposed LF module improves the detection of fine-grained lesions by combining lightweight channel attention with cross-layer feature fusion. This design alleviates the common issue of small target information loss in conventional approaches. (2) The DDC module dynamically adjusts the receptive field through dilated convolutions with multiple dilation rates and adaptive weight allocation. Through this mechanism, the model becomes more adept at identifying lesions with irregular morphology. (3) The LA module guides the network to focus on potential lesion regions using a region-based partition strategy. It also suppresses background noise through local context modeling, thereby improving the model’s edge perception and lesion discrimination. Collectively, these modules contribute to a robust and efficient model that supports intelligent plant disease diagnosis in real-world agricultural settings.

Experimental results demonstrate that the proposed LDL-MobileNetV3S model significantly enhances the recognition performance for potato leaf diseases while maintaining a lightweight architecture. Compared with existing lightweight models, it achieves superior results in key evaluation metrics such as accuracy and recall. These outcomes validate the effectiveness of the multi-module fusion strategy in lightweight neural networks and offer a practical and scalable solution for real-time plant disease diagnosis in agricultural edge computing scenarios.

## 2 Materials and methods

### 2.1 Data and processing

#### 2.1.1 Dataset

The dataset employed in this study comprises two primary components: publicly available data and self-acquired data. The majority of the public data were sourced from the PlantVillage platform. The dataset comprised 2400 images of potato leaves gathered under field conditions. These images were predominantly captured under controlled conditions (e.g., consistent lighting and background), resulting in high image quality and clarity. Such controlled environments facilitate the extraction of robust training features for the model. In August 2024, the research team conducted the field component of the self-acquisition process at the Xufeng Potato Experimental Base in Wuchuan County, Hohhot City. Utilizing a Huawei Mate 60 smartphone, they captured images of 2348 instances of potato leaf diseases. This subset of images, which accurately captured field environmental elements such as natural lighting, complex backgrounds, and leaf shading, enhanced the model’s ability to adapt to complex real-world scenarios.

**Figure 1:**
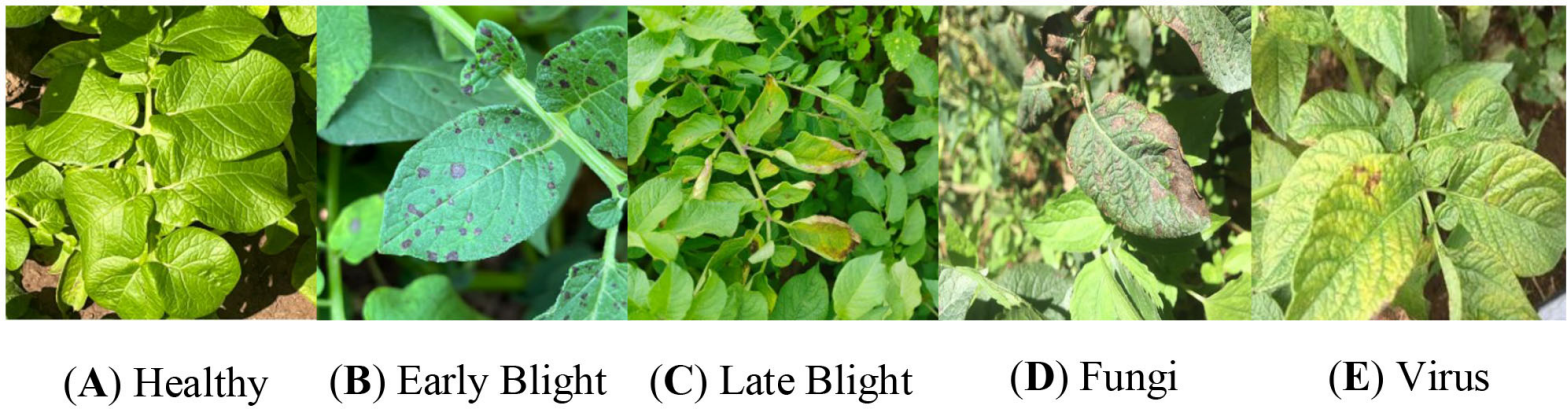
Sample images of potato leaf diseases. **(A)** Healthy, **(B)** Early Blight, **(C)** Late Blight, **(D)** Fungi, **(E)** Virus.

All photographic samples were subjected to rigorous screening and preprocessing to eliminate instances of blurring, duplication, and poor clarity, thereby enhancing the overall quality of the dataset. The final dataset comprises a total of 4748 potato leaf images, encompassing five distinct categories: healthy leaves and four types of diseases. Representative samples for each category are illustrated in **Figure 1**. To ensure a robust and stable model training process, the dataset was split using a single fixed stratified partition with an 8:1:1 ratio for training, validation, and testing sets. This partitioning strategy also ensured that the categories were evenly distributed across the subsets, thereby mitigating the potential impact of class imbalance on model performance. The specific distribution of the data is detailed in **Table 1**.

**Table 1:** Proportional split of potato leaf dataset.

Split	Healthy	Early blight	Late blight	Fungi	Virus	Sum
Train	800	800	800	608	800	3808
Validation	100	100	100	70	100	470
Test	100	100	100	70	100	470

#### 2.1.2 Data augmentation and preprocessing

Due to the limited number of samples in the potato leaf disease dataset, training a deep neural network remains difficult. Most of the available data originate from controlled laboratory settings, while samples from natural field environments are insufficient. This imbalance restricts the model’s generalization ability in real-world applications. To address this limitation, this study proposes a systematic data augmentation strategy. The method enhances training diversity by simulating various image variations typically encountered in complex field conditions.

**Figure 2:**
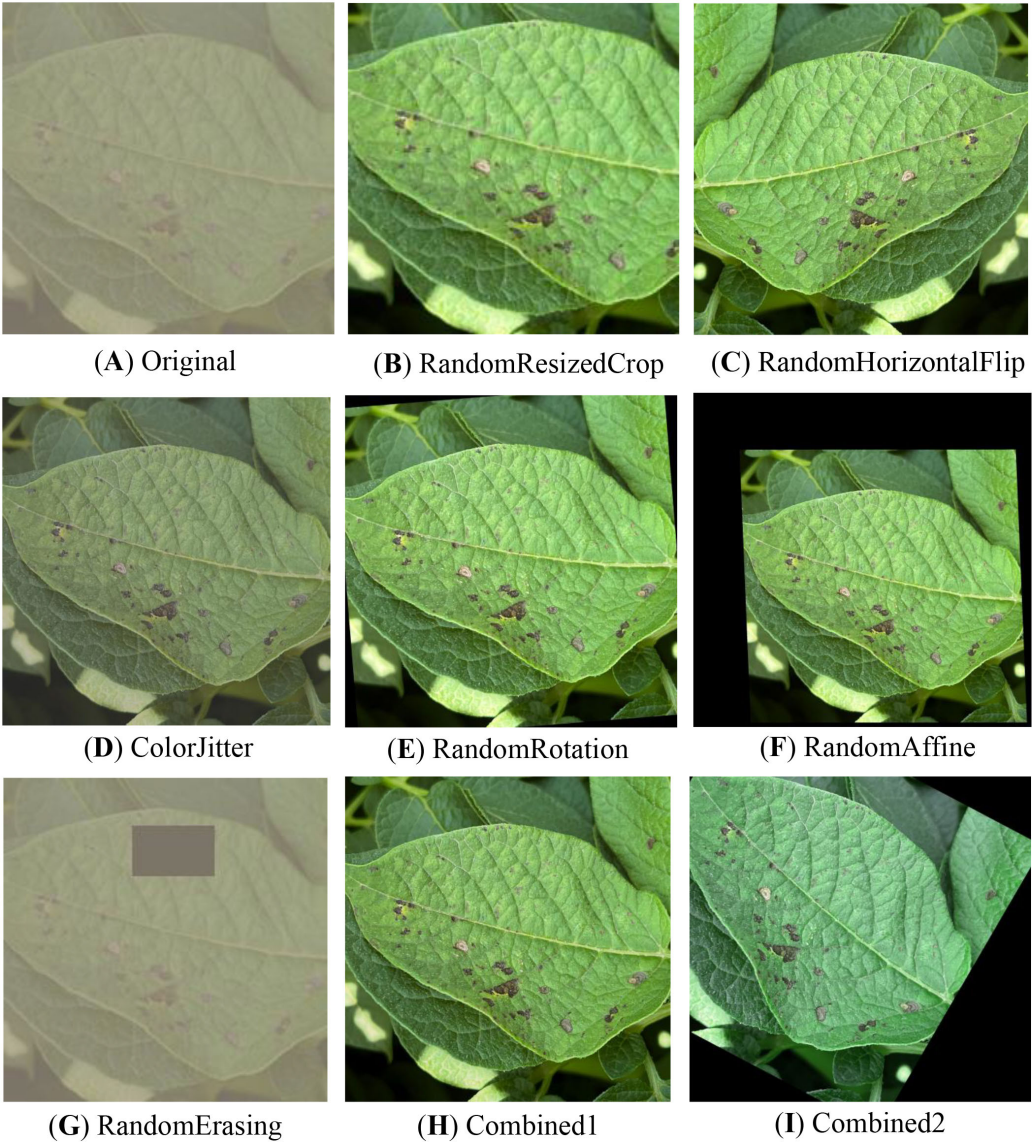
Data augmentation results. **(A)** Original, **(B)** RandomResizedCrop, **(C)** RandomHorizontalFlip, **(D)** ColorJitter, **(E)** RandomRotation, **(F)** RandomAffine, **(G)** RandomErasing, **(H)** Combined1, **(I)** Combined2.

The data augmentation technique employed in this study consists of four essential components, the effects of which are illustrated in **Figure 2**. Initially, the ColorJitter operation is utilized to randomly adjust the brightness, contrast, and other attributes of the images. The objective is to emulate the fluctuating illumination often present in uncontrolled agricultural environments. Subsequently, a series of geometric transformations, including RandomRotation, RandomHorizontalFlip, RandomVerticalFlip, and RandomAffine, are applied to introduce spatial variations. These transformations enhance the feature representation of leaves in diverse orientations and angles, thereby improving the robustness of the model to different leaf poses and viewing perspectives. To simulate the common occurrences of leaf breakage and occlusion in real-world scenarios, the RandomErasing technique is employed to randomly erase a portion of the image. This method introduces variability in the data by simulating partial missing regions, which enhances the model’s robustness to incomplete or obstructed leaf images. Furthermore, the RandomResizedCrop operation is utilized to perform random cropping and resizing of the images. This not only increases the diversity of image perspectives and compositions but also helps in augmenting the dataset by generating additional variations of the leaf images.

To ensure the stability of the training process and the generalizability of subsequent model transfer, all augmented images were uniformly resized to 224×224 pixels and normalized using the mean and standard deviation values from the ImageNet dataset. Two composite data augmentation schemes were devised to further investigate the impact of various enhancement strategies on model performance. The first scheme, Combined1, integrates luminance adjustment and horizontal flipping to simulate variations in structural orientation and illumination conditions. The second scheme, Combined2, combines random rotation with contrast adjustment to enhance the model’s robustness to angular changes and color perturbations. The proposed schemes serve to examine the contribution of diverse augmentation approaches to improving model generalization and accuracy.

In the primary experiments (including ablation and comparative studies), we employed a unified data augmentation pipeline, in which the aforementioned augmentation techniques were sequentially combined to form a fixed process, thereby effectively enhancing the diversity of the training data. The two composite augmentation strategies (Combined1 and Combined2) were only applied in supplementary comparison experiments to explore the impact of different augmentation combinations on model performance and were not part of the default training pipeline. During validation and standard testing, only image resizing and normalization were applied to ensure fairness in evaluation. For the final model evaluation, test-time augmentation (TTA) was introduced, whereby multiple views of each sample (including flips, rotations, and color perturbations) were generated and their predictions averaged, in order to further improve the stability of the evaluation process.

### 2.2 Introduction to the MobileNetV3 Small network architecture

Google unveiled MobileNetV3, a small and effective deep neural system designed for situations with limited resources like embedded and mobile gadgets in 2019 (Wang et al., 2020). The architectural principles of MobileNetV1 and V2 are extended and refined in MobileNetV3 Small, which is specifically designed for mobile scenarios with limited processing resources (Zhao and Wang, 2022). Owing to its compact structural design and favorable balance between accuracy and efficiency, MobileNetV3 Small emerges as a highly competitive candidate among various lightweight neural network models. The Small version of MobileNetV3 is particularly well-suited for deployment on end devices that have limited computational power and are subject to power consumption constraints. Compared to its Large counterpart, MobileNetV3 Small exhibits significant advantages in terms of model size and inference time (Qian et al., 2021). In the context of engineering deployment, MobileNetV3 Small offers greater flexibility and convenience. Unlike other lightweight networks such as ShuffleNet, EfficientNet, or Tiny-YOLO (Redmon and Farhadi, 2017) (Redmon and Farhadi, 2018), it maintains a robust capability for image feature extraction while effectively compressing the number of parameters.

**Figure 3:**
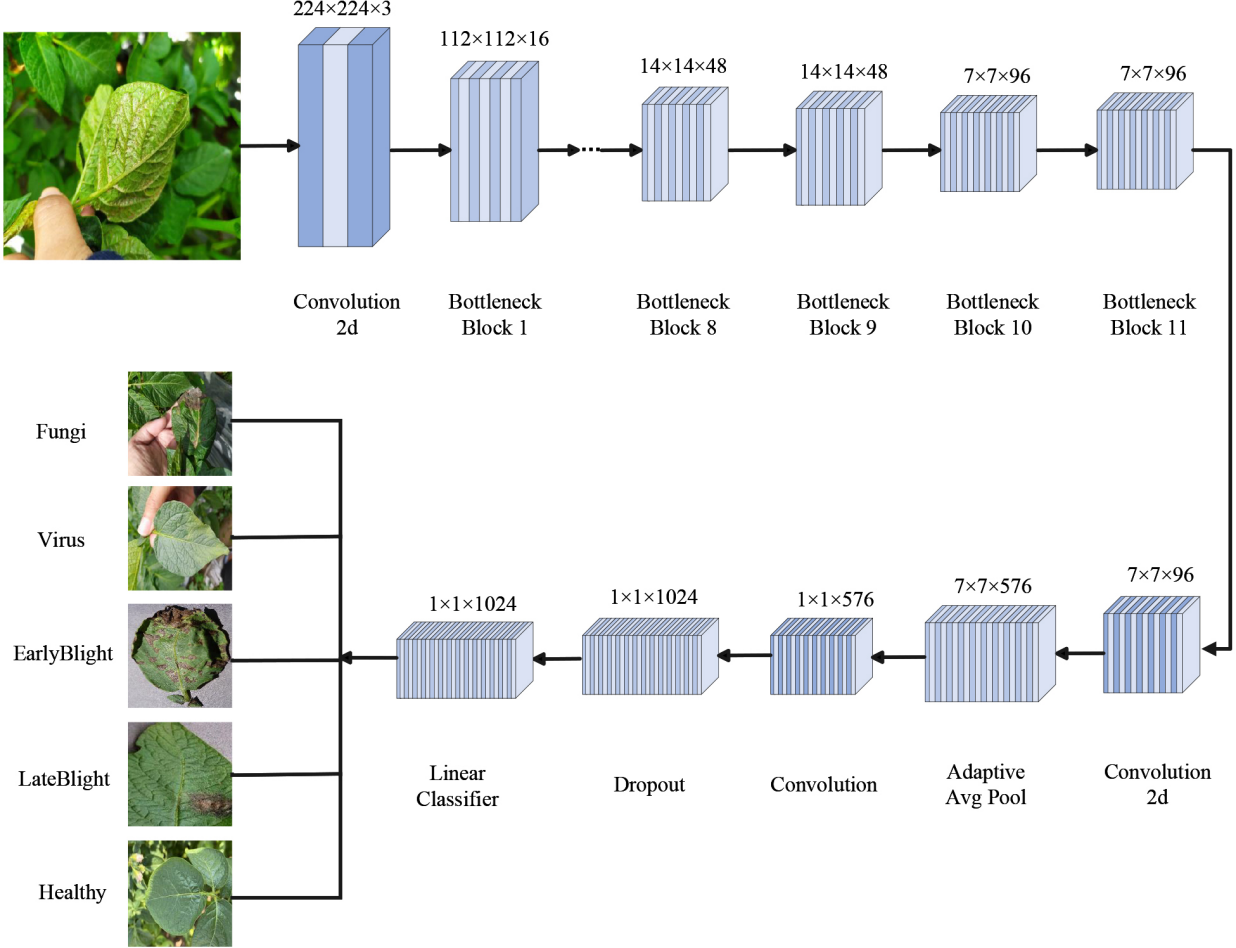
Architecture of the MobileNetV3S model.

The task of crop disease detection necessitates a model capable of real-time operation on mobile terminals or edge devices, in addition to possessing robust identification capabilities (Jiang and Li, 2020). As illustrated in **Figure 3**, MobileNetV3 Small is selected as the underlying network architecture in this study. This choice is primarily driven by the actual deployment environment, which predominantly consists of field sites where devices often face challenges such as limited computational power, insufficient power supply, and stringent response time requirements.

The MobileNetV3 Small model employs a lightweight deep neural network architecture. It begins with an input image of size 224×224×3. A 3×3 convolutional layer is first applied for feature extraction and downsampling, reducing the resolution to 112×112×16. This is followed by 11 sequentially stacked Inverted Residual Bottleneck modules. These modules use different convolutional kernel sizes, such as 3×3 and 5×5, depending on the stage. They apply varying channel expansion ratios. Each module may also incorporate the SE attention mechanism and use either the ReLU or HSwish activation function. During the process, the feature map is gradually reduced to a size of 7×7×96. A 1×1 convolution is then applied to expand the channels to 576. Global average pooling is used to summarize spatial features. A feature projection layer, also using 1×1 convolution, generates a 1024-dimensional vector. Finally, a fully connected layer produces the classification results. This structure is well-suited for image recognition tasks on mobile and edge devices, as it balances model accuracy with computational efficiency.

### 2.3 The LDL-MobileNetV3S classification model for potato leaf diseases

Despite its good lightweight qualities for mobile deployment and edge computing capabilities, MobileNetV3 Small still has certain limitations when it comes to processing images of potato leaf disease in complex agricultural settings. For instance, the model is weak in capturing local fine-grained lesion features due to its insufficient feature expression capabilities, which reduces overall classification accuracy. Potato diseases manifest in real photos in a variety of forms and sizes, and the design’s limited capacity to adapt to illnesses at various scales makes it difficult to establish an efficient multi-scale feature distribution, thereby lowering recognition performance. Lastly, due to the model’s lack of a mechanism to focus on particular locations, it can be challenging to accurately reference the distinct features of the diseased space, this is vulnerable to confusion between categories.

**Figure 4:**
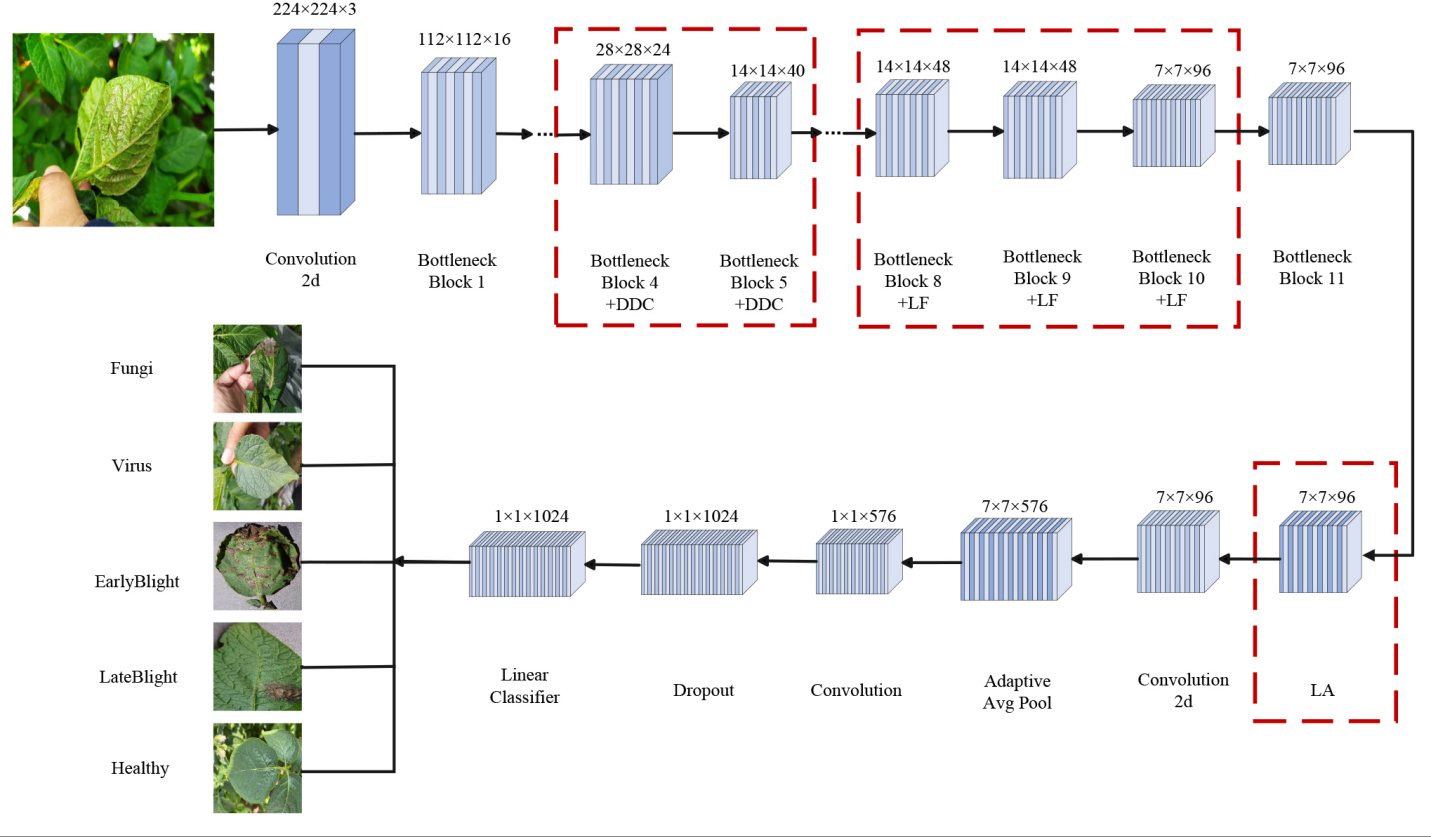
Architecture of the LDL-MobileNetV3S model.

To overcome the stated limitations, this work presents a lightweight architecture that extends MobileNetV3 Small through targeted structural modifications. The improved MobileNetV3 Small model uses standard convolution and multi-layer Bottleneck blocks to extract features. It integrates the Dynamic Dilated Convolution module to enhance multi-scale perception and adds the Lite Fusion module to fuse high-level and low-level features, improving spatial detail representation. The Lightweight Attention module then highlights key information. Finally, global pooling, feature projection, and fully connected layers complete the classification. This design boosts recognition accuracy and feature expression while keeping the model lightweight. **Figure 4** shows the entire workflow.

Three specialized enhancement modules are proposed and incorporated at critical points within the backbone feature extraction stage.

a. Lite Fusion module

To extract information from high-resolution shallow features, the module Lite Fusion is inserted after layers 8, 9, and 10 of the Inverted Residual Bottleneck. These features are channel-enhanced by the SE attention mechanism, downsampled by a 1×1 convolution, and then concatenated with the deeper features of the current layer. The LiteFusion module facilitates cross-layer feature fusion, which effectively addresses the problem of information degradation across network layers. As a result, it substantially enhances the model’s capability to capture fine-grained features, thus enabling the network to more accurately localize and classify small lesions (Chen et al., 2018) (Lin et al., 2017).

b. Dynamic Dilated Convolution module

In the 4th and 5th Inverted Residual Bottleneck, the standard Depthwise Convolution is replaced with Dynamic Dilated Convolution. This module creates three convolution branches with varying dilation rates (1, 3, and 5) and combines their outputs using attention-based weighting for dynamic receptive field modeling. The DynamicDilatedConv module integrates the principles of Dilated Convolution (Yu and Koltun, 2015) and Dynamic Convolution mechanisms (Chen et al., 2020). It adaptively adjusts the receptive field size to capture lesion features at varying scales. This design significantly enhances the model’s ability to identify diverse lesion regions in complex agricultural images. It is particularly effective for detecting lesions with blurred boundaries, small sizes, irregular shapes, or varying diffusion patterns. The dynamic adaptation mechanism allows the model to better address common challenges in real-world scenarios, such as scale variation and uneven lesion spread.

c. Lightweight Attention module

To improve the model’s localization and recognition accuracy under complex backgrounds, insert Lightweight Attention after the last Inverted Residual Bottleneck. This module divides the feature map into multiple fixed windows and applies the QKV self-attention mechanism within each window to highlight the diseased spot region and enhance the local structure modeling ability.

Lastly, the classifier module receives the enhanced higher-order semantic features. The module employs Softmax to classify five different potato leaf conditions and consists of Global Average Pooling (GAP), a Dropout Layer, and a Fully Connected Layer.

Utilizing the model parameters presented in **Table 2**. Experimental evaluation was performed using a specialized image set focused on potato leaf pathology.

**Table 2:** Parameter settings of the LDL-MobileNetV3S model.

Input size	Operation	Expsize	Output channels	ICA/SE	Activation	Stride
224×224×3	Conv2d, 3×3	–	16	–	HSwish	2
112×112×16	Bottleneck, 3×3	16	16	✓	ReLU	2
56×56×16	Bottleneck, 3×3	72	24	–	ReLU	2
28×28×24	Bottleneck, 3×3	88	24	✓	ReLU	1
28×28×24	Bottleneck (DynamicDilated), 5×5	96	40	✓	HSwish	2
14×14×40	Bottleneck (DynamicDilated), 5×5	240	40	✓	HSwish	1
14×14×40	Bottleneck, 5×5	240	40	✓	HSwish	1
14×14×40	Bottleneck, 5×5	120	48	✓	HSwish	1
14×14×48	Bottleneck, 5×5	144	48	✓	HSwish	1
14×14×48	Bottleneck, 5×5	288	96	✓	HSwish	2
7×7×96	Bottleneck, 5×5	576	96	✓	HSwish	1
7×7×96	Bottleneck, 5×5	576	96	✓	HSwish	1
–	LiteFusion Module #1	–	+Concat	✓	HSwish	Upsample×2
–	LiteFusion Module #2	–	+Concat	✓	HSwish	Upsample×2
–	LiteFusion Module #3	–	+Concat	✓	HSwish	Upsample×2
7×7×96	Lightweight Attention	–	96	✓	HSwish	–
7×7×96	Conv2d, 1×1	–	576	–	HSwish	1
7×7×576	Adaptive Avg Pool	–	576	–	–	–
1×1×576	1×1 Conv (Feature projection)	–	1024	–	HSwish	–
1×1×1024	Dropout (p=0.2)	–	1024	–	–	–
1×1×1024	Fully Connected	–	num_classes	–	Softmax	–

#### 2.3.1 Lite Fusion

Using the concept of Feature Pyramid Network (FPN) and merging the properties of MobileNetV3 Small lightweight structure, this study proposes a feasible LF fusion proximity.

**Figure 5:**
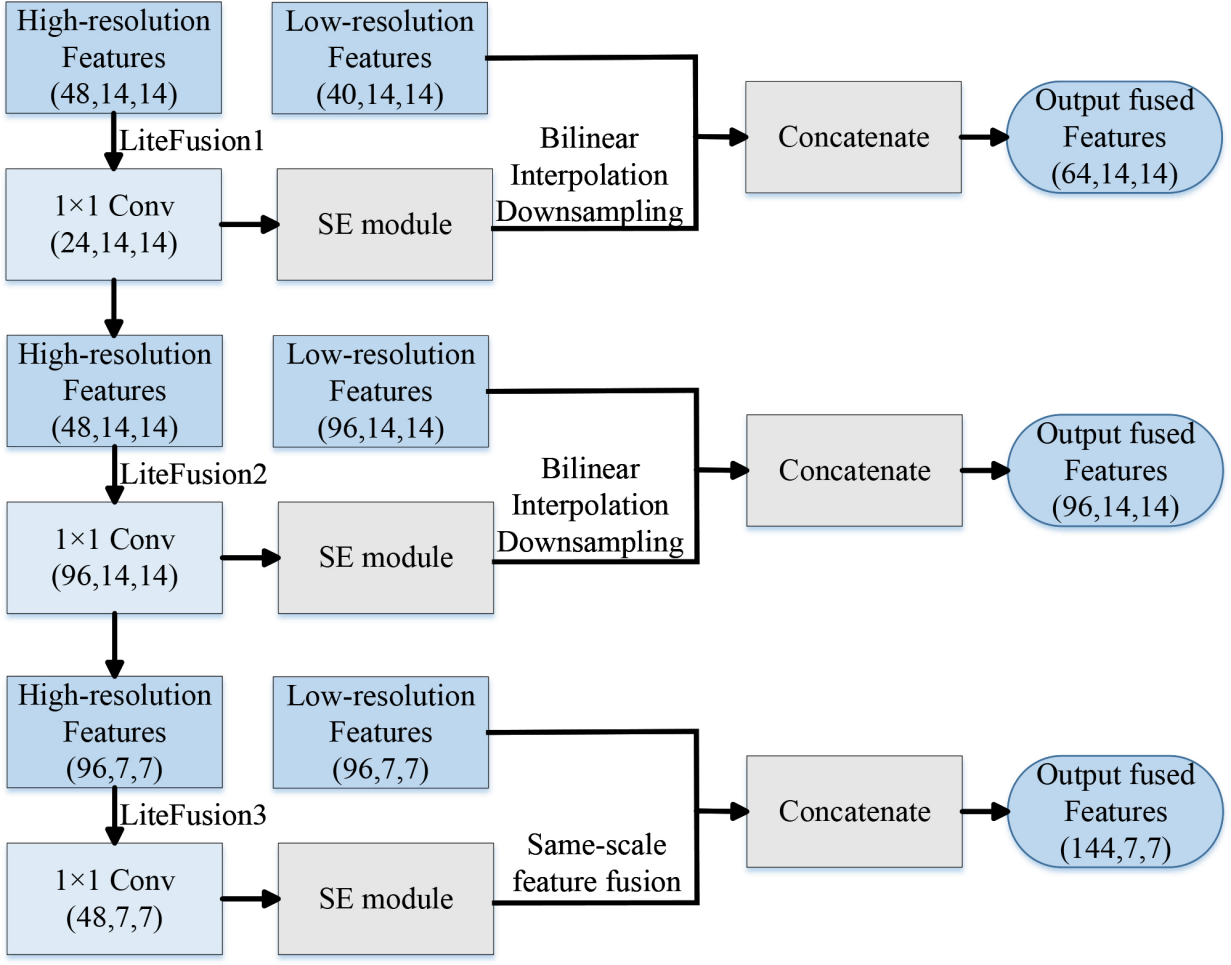
Structural flow diagram of the LF module in the LDL-MobileNetV3S model.

In the disease recognition task, the design of the LF module is critical to boost the functionality of models. It receives low-resolution features from deeper layers, which contain rich semantic information, and high-resolution features from shallower layers, which preserve edge and texture details. The structure is illustrated in **Figure 5**. To reduce computational costs and match dimensionality, the module first applies channel compression to the high-resolution features using a 1×1 convolution. These characteristics are then weighted using the channel’s focus approach. After that, the high-resolution feature maps undergo bilinear interpolation downsampling to match the size of the low-resolution feature maps. Ultimately, the fused multi-scale semantic features are produced by concatenating the low-resolution features with the compressed high-resolution features in the channel dimension.

The module combines the low-resolution and high-resolution feature maps, enabling the model to make use of either worldwide and local semantic data to better identify the characteristics of potato leaf diseases. Its precise calculation procedure is as follows:

The high-resolution feature map $X\in R^{B\times C\times H\times W}$ is first supplied into the SE module, where B denotes batch size, C for channel count, and H×W for input feature map spatial dimensions (W stands for width, and H for height). The channel attention method primarily uses the feature vector X, which is computed as shown by (Equations 1–3), to reduce the unimportant inputs in order to improve the expression of the traits and concentrate on more of the important feature channels.


(1) $$S_{c}=\frac{1}{H\times W}\sum_{i=1}^{H}\sum_{j=1}^{W}X_{c}\left(i,j\right)$$

(2) $$e_{c}=\sigma \left(W_{2}\delta \left(W_{1}S_{c}\right)\right)$$

(3) $$X_{c}^{\prime }=e_{c}\cdot X_{c}$$

With *S_c_* standing for the Squeeze output result for the c-th channel, which represents the response strength of the global average for that channel, (Equation 1) determines the average value for each channel by global average pooling. The intrinsic value of the c-th channel of the given input characteristic map at spatial point (i,j) is denoted by the symbol *X_c_* (i,j). (Equation 2) dynamically learns the weights of each channel, where *e_c_* is the channel attention weight, *W*_1_ is the dimensional reduction FC, which serves to minimize computational effort, *W*_2_ is the dimensional enhancement FC, which restores the original dimensional, *δ* is the function that activates the ReLU, while *σ* is the Sigmoid normalization. Channel weighting is shown in (Equation 3) to improve the response of the key channels.

Second, 1×1 convolution’s channel compression improves cross-channel information interaction while lowering computation. (Equations 4, 5), respectively, display the computation channel-by-channel formulas and the total convolution operation:


(4) $$X^{\prime }=W*X$$

(5) $$X_{c^{\prime }}^{\prime }=\sum_{c=1}^{c}W_{c^{\prime },c}X_{c}$$

where $W_{c^{\prime },c}$ is the scalar weight in the weight matrix that joins the input channel c to the output channel ${\text{c}}^{\prime }$ , and $X_{c^{\prime }}^{\prime }$ indicates the $c^{\prime }$ channel in the consequence feature vector.

Since the size of the high resolution feature differs from that of the trait of low resolution, the high resolution feature is downsampled, and its spatial dimension is changed to match that of the functionality for low resolution, per (Equation 6):


(6) $$X^{\prime \prime }\left(i,j\right)=\sum \;\sum \;W_{m,n}X^{\prime }\left(m,n\right)$$

The feature map X" is the result of channel compression, and the bilinear interpolation weights are *W_m,n_*. One of the parameter-free operations, bilinear interpolation may successfully decrease the amount of data on location lost and offers the benefits of easy implementation, quick computation and a smooth transition.

Lastly, channel dimension splicing is performed, and **Table 3** displays the splicing dimensions. After channel compression and downsampling, the fused features contain both low-resolution and high-resolution features. The spliced features can then be fed into the deep network for higher-level learning to improve the multi-scale feature identification capabilities.

**Table 3:** Input and output dimensions of the LF modules in the LDL-MobileNetV3S model.

Module name	Insertion position	High-resolution input	Low-resolution input	Output of fused features
LiteFusion1	After 8th Bottleneck	(48, 14, 14)	(40, 14, 14)	(64, 14, 14)
LiteFusion2	After 9th Bottleneck	(48, 14, 14)	(96, 14, 14)	(96, 14, 14)
LiteFusion3	After 10th Bottleneck	(96, 7, 7)	(96, 7, 7)	(144, 7, 7)

#### 2.3.2 Dynamic Dilated Convolution

In deep learning tasks for image classification and target detection, CNNs typically perform feature extraction from input images using a fixed-size convolution kernel. However, the convolution structure with fixed receptive fields has limitations in handling visual targets with significant scale variations. This is particularly evident in the task of detecting crop diseases in complex backgrounds, where lesions vary greatly in morphology, size, and density. A single-scale convolution kernel finds it difficult to strike a balance between broad semantic details and local specifics, therefore hurting the system’s precision and resilience.

**Figure 6:**
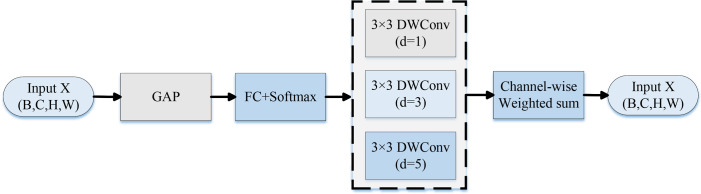
Structural flow diagram of the DDC module in the LDL-MobileNetV3S model.

DDC is an effective method to enhance the receptive field modeling capability of CNNs. Traditional dilated convolution expands the receptive field by introducing interval expansion in the convolution kernel to obtain more contextual information without increasing the number of parameters. However, its fixed-dilation-rate design lacks flexibility to accommodate different scale objectives. DDC dynamically adjusts the range of receptive field response by modeling the feature map at multiple scales using multiple convolution kernels with different dilation rates in parallel and implementing a channel attention system to adapted weight each branch’s outputs for fusion, as shown in **Figure 6** below.

This strategy effectively distinguishes similar disorders, like early blight (sharp edges) and late blight (fuzzy edges), in the early stage and improves detection performance in complex scenarios such as blurred spot contours and variable scales. The precise method of calculation will be as follows:

Before calculating the channel attention, the features X (B, C, H, W) are first input into the DDC for feature extraction. The channel attention mechanism uses global information to adaptively assign weights with varying expansion rates by computing the global average pooling (GAP). This allows the network to dynamically modify the receptive field according to an input picture. The formulas are given in (Equations 7, 8):


(7) $$F_{gap}=\frac{1}{H\times W}\sum_{i=1}^{H}\sum_{j=1}^{W}X\left(i,j\right)$$

(8) $$W=Softmax\left(Linear\left(F_{gap}\right)\right)$$

Then, employing multiple expansion rates d (e.g., 1, 3, 5) for the extraction of attributes at various scales, several parallel 3 × 3 depth-separable convolutions are built, which helps capture more discriminative patterns in affected zones, thereby enhancing the model’s decision-making ability. The formula is shown in (Equation 9):


(9) $$F_{i}=X*K_{i}$$

where *d_i_* is a convolution kernel that measures 3 × 3 and *K_i_* is the expansion rate; each channel is calculated manually to avert over-computing.

The final feature Fout(B, C, H, W), which aggregates local and global data to enhance the robustness of illness diagnosis, is then produced using dynamic weighted summation. The formula for the calculation is displayed in (Equation 10):


(10) $$F_{out}=\sum_{i=1}^{N}W_{i}\cdot F_{i}$$

With *W_i_* representing the attention weight and *F_i_* representing the output of several expansion rate convolutions.

In an effort to improve the accuracy of detecting potato diseases, this module can broaden the model’s field of perception to concentrate on both local and global disease aspects. **Table 4** depicts the precise insertion positions as well as the input and output dimensions.

**Table 4:** Input and output dimensions of the DDC module in the LDL-MobileNetV3S model.

Insertion position	Input X	GAP output	FC + softmax output	3×3 conv output	Output after fusion
Level 4	(B, 96, 28, 28)	(B, 96, 1, 1)	(B, 3)	(B, 96, 28, 28)	(B, 96, 28, 28)
Level 5	(B, 160, 28, 28)	(B, 160, 1, 1)	(B, 3)	(B, 160, 28, 28)	(B, 160, 28, 28)

#### 2.3.3 Lightweight Attention

Computer vision applications including target recognition, image segmentation and image classification have made extensive use of the attention mechanism in deep learning models (Ghaffarian et al., 2021). Specifically, the image’s global dependencies might be better captured by the system for self-attention by modeling the correlation between various locations within the features, which improves the model’s feature extraction and image recognition (Hu et al., 2023).

**Figure 7:**
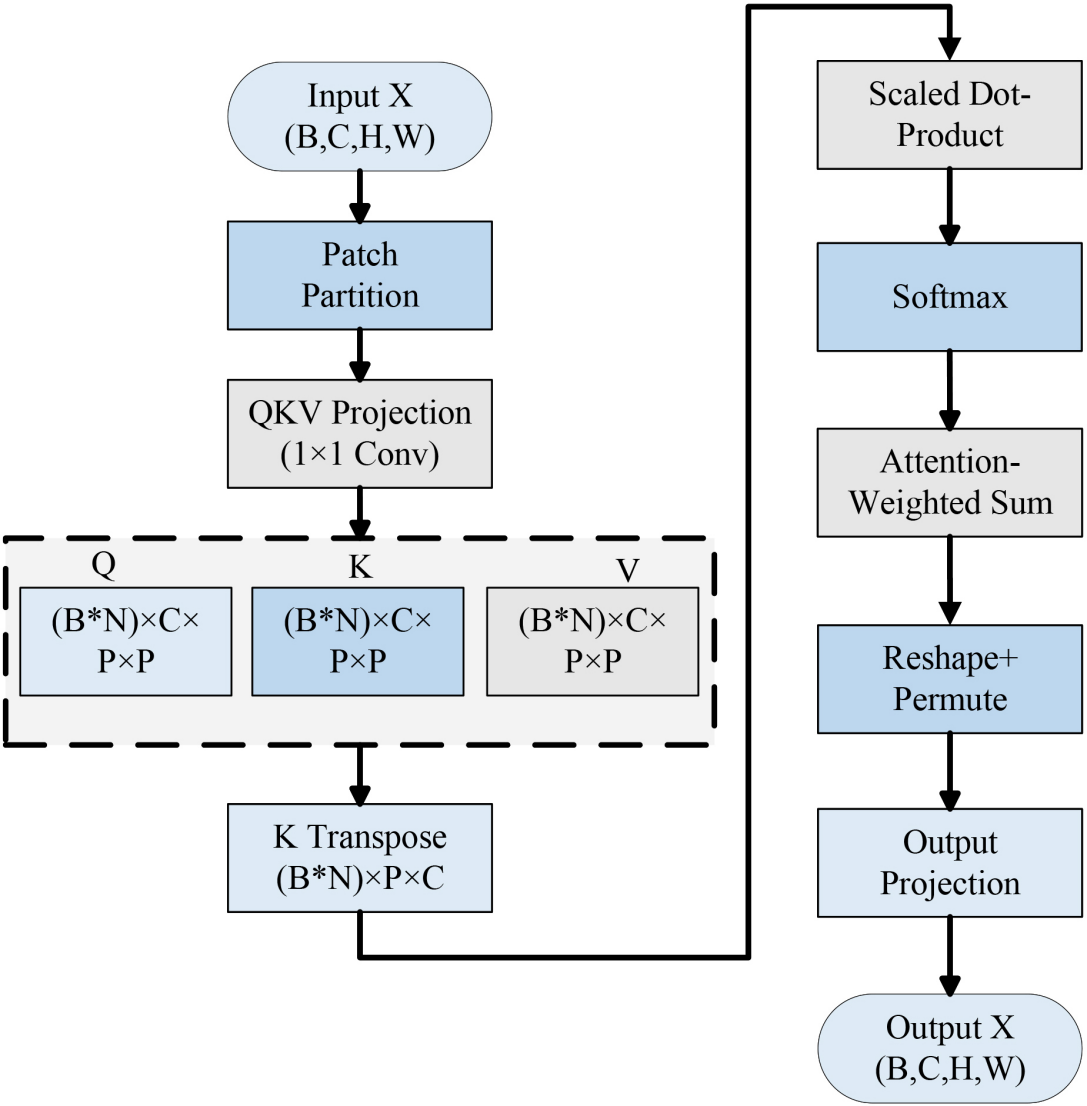
Structural flow diagram of the LA module in the LDL-MobileNetV3S model.

The typical self-attention structure offers multiple advantages when it comes to modeling global information. However, its application in mobile and edge devices is complicated by its high computational cost and numerous parameters. More lightweight and real-time models are needed for disease detection systems in agricultural contexts, they usually run on hardware with little computing power (like drones, farm terminals, or mobile devices). Therefore, the current study introduces a lightweight LA module at the backend of the MobileNetV3 Small model. This module increases the potential of the network to concentrate on sick regions without significantly increasing the model’s computational load, as depicted in **Figure 7**.

This module expands the capacity of the model to localize the target spots and suppress irrelevant backgrounds through mutual feature modeling and local window partitioning. As a result, it enhances the overall classification accuracy and robustness. The following is the precise formulating process:

Split the input feature $X\in R^{B\times C\times H\times W}$ into a P×P patch: $X\to X_{patche}\in R^{B\times C\times \frac{H}{P}\times P\times \frac{W}{P}\times P}$ . Next, the reorganization of the dimensions: $X_{patch}\to X_{flat}\in R^{\left(B\times \frac{H}{P}\times \frac{W}{P}\right)\times C\times P\times P}$ , In this manner, each patch can be calculated separately.

The Query, Key, and Value (QKV) representations are generated using a shared 1×1 convolution, and the output is subsequently split into three separate components. Query and transposed key scaling and matrix multiplication are used for calculating the attention score. Softmax normalization is then used to obtain the attention weights in (Equation 11):


(11) $$Attention\left(Q,K\right)=Softmax\left(\frac{QK^{T}}{\sqrt{C}}\right)$$

After calculating the final feature *X_attn_* = *Attention* × *V*, Value is weighted and added to the weights that were determined in order to accomplish feature aggregation. The original size of the feature map is then restored through transpose and reshape operations. Finally, using a convolution with a 1x1 projection to produce an output with the same spatial dimensions as the input. Through parameter sharing and localized attention methods, the module drastically decreases computational complexity while still preserving the attention mechanism’s primary benefits that are making it suitable for effective integration into CNN systems.

### 2.4 Model evaluation metrics

The effectiveness of the modified LDL-MobileNetV3S model in the potato leaf disease classification task is quantitatively analyzed in this paper using a range of evaluation metrics, including incorporating standard classification measures like F1-score, Accuracy, Precision, and Recall. These measurements are employed to meticulously and impartially assess the system’s functionality. Additionally, a confusion matrix is employed for fine-grained misclassification analysis, with the goal of offering a thorough summary of the efficiency of the model on both a general and specific level. The following are the formulas and meanings of these metrics:

From the perspective of classification modeling, accuracy is a frequently utilized measure for assessment. It indicates the proportion of samples that have been correctly classified out of the entire sample population. The computational process is defined in (Equation 12):


(12) $$Accuracy=\frac{TP+TN}{TP+TN+FP+FN}$$

In this context, the term TP (True Positives) denotes the quantity of samples that the model accurately classified as diseased. The total quantity sample size that the model reliably classified as healthy leaves is known as TN (True Negatives). A sample’s FP (False Positives) reflects the number of instances where healthy leaves were mistakenly identified as diseased by the model. And FN (False Negatives) is the quantity of samples in which the model incorrectly categorized diseased leaves as either healthy or falling into a different group.

The precision rate, which indicates the percentage of genuine diseased leaves among all the leaves predicted to be infected by a certain disease, calculates the percentage of samples in a given category that the model actually predicts to be positive. (Equation 13) shows the formula for calculating precision.


(13) $$Precision=\frac{TP}{TP+FP}$$

For disease control, high precision in disease detection tasks means that the model is less likely to generate false alarms when predicting a specific disease. This is of symbolic significance, as it indicates that fewer healthy leaves are incorrectly recognized as infected.

Recall is the percentage of all leaves that are truly plagued with a particular condition and are correctly identified, indicating the model’s capacity to detect diseased leaves. (Equation 14) presents its computation formula:


(14) $$Recall=\frac{TP}{TP+FN}$$

Enhancing the recall rate is crucial for obtaining early warnings and implementing precise disease prevention. It is an essential factor in ensuring agricultural safety and promoting the development of smart agriculture. The level of the recall rate directly affects the detection coverage of diseases.

The F1-score, regarded as the equilibrium value of recall and precision, serves as a comprehensive tool to evaluate how well these two measures are balanced. Its calculation is provided in (Equation 15):


(15) $$F1-score=2\times \frac{Precision\times Recall}{Precision+Recall}$$

Within this research, when assessing the enhanced MobileNetV3 Small model’s functionality, we emphasized not only classification accuracy but also the F1-score. This dual focus ensures that the model minimizes the misclassification of healthy leaves as diseased while maximizing the detection of all diseased leaves in practical applications. The ultimate goal is to provide efficient and reliable support for disease identification, prevention, and control.

## 3 Results and analysis

### 3.1 Ablation study

This study was implemented using the following computational and software resources: an Intel(R) Core(TM) i5-8300H CPU operating at 2.30 GHz, equipped with 32 GB of DDR4–2667 compute memory, running on a 64-bit Microsoft Windows 10 operating system. The model building and training were performed using the PyTorch 2.6.0+cpu deep learning framework within a Python 3.11 environment. This paper’s ablation studies are intended to confirm the efficacy of the LF, DDC, and LA modules for MobileNetV3 Small in potato leaf disease detection tasks. The standard MobileNetV3 Small (S0) is used as the baseline model. Based on this, different improvement modules are introduced respectively, and each one module’s effect on the model’s functionality is examined. **Table 5** displays the findings of the experiment.

**Table 5:** Ablation study results of the LDL-MobileNetV3S model.

Schema	Base	LF	DDC	L	Loss	Accuracy/%	Precision/%	Recall/%	F1 score/%
S0	✓				0.279	88.51	87.17	86.64	86.57
S1	✓	✓			0.022	90.21	89.63	89.28	89.19
S2	✓		✓		0.022	90.64	89.93	89.38	89.41
S3	✓			✓	0.022	90.85	90.11	89.66	89.61
S4	✓	✓		✓	0.023	89.36	88.60	88.23	88.13
S5	✓	✓	✓		0.021	91.06	89.89	89.67	89.65
S6	✓		✓	✓	0.022	93.40	93.25	92.29	92.51
S7	✓	✓	✓	✓	0.020	94.89	93.54	92.53	92.77

The baseline scenario (S0) obtained an F1 score of 86.57%, an accuracy of 88.51%, and a loss value of 0.279 on the test set using the MobileNetV3 Small model for potato leaf disease detection without any improvement modules. This suggests that the standard MobileNetV3 Small is still effective for extracting disease features in this task, but there is room for improvement. To ascertain how each part affects the model’s functionality, this study adds the LF, DDC, and LA modules to the baseline model for testing. The LF module in S1 (S0 + LF) aims to maximize the recognition ability of disease areas at various scales and improve the information interaction across different feature layers. The verification results show that the module increases the accuracy to 90.21%, the F1 score to 89.19%, and reduces the loss value to 0.022. This indicates that LF can significantly enhance the model’s feature extraction capabilities. By dynamically modifying the accepting field of widened convolution, the DDC module in configuration S2 (S0 + DDC) improves the strategy’s capacity to adapt to varying sick section sizes. The experimental results demonstrate the success of DDC in capturing multi-scale disease characteristics, with the module increasing the accuracy to 90.64%, the F1 score to 89.41%, and reducing the loss value to 0.022. The LA module in S3 (S0 + LA) reduces computational overhead while enhancing the method’s focus on the disease region. The experimental results confirm the module’s key role in disease identification, showing that it improves the accuracy to 90.85%, the F1 score to 89.61%, and reduces the loss value to 0.022.

This article evaluates the performance of multi-module combinations to further analyze the synergies between various modules. By incorporating the LF and LA modules, S4 (S0 + LF + LA) improves accuracy to 89.36%, the F1 score to 88.13%, and reduces the loss value to 0.023. The above results show that there is still room for development in this combination’s feature extraction capabilities. When the LF and DDC modules are combined in S5 (S0 + LF + DDC), the accuracy increases to 91.06%, the F1 score improves to 89.65%, and the loss value drops to 0.021. This demonstrates that this combination can effectively enhance the model’s adaptability to the disease area. S6 (S0 + DDC + LA) combines the DDC and LA modules and achieves significant improvement in most metrics, with 93.40% accuracy, 92.51% F1 score and a loss value of 0.022. This suggests that DDC and LA have a strong complementary effect that can help improve the model’s feature extraction capability. Based on the S6 scheme, S7 (S0 + LF + DDC + LA) adds the LF module. The outcomes of the studies demonstrate that this plan performed the best across the board, with an accuracy of 94.89%, an F1 score of 92.77%, and a loss value of 0.020. This indicates that the integration of all three modules can improve classification performance, reduce the loss value, and significantly enhance the ability to detect potato leaf disease.

Based on findings of the experiment, adding the LF, DDC, and LA modules individually can improve the disease detection performance of MobileNetV3 Small. The DDC module shows the most significant improvement, indicating that the DDC enhancement method can more effectively increase the model’s adaptability to disease areas at various scales. By merging several modules, the model’s efficacy can be further enhanced; in particular, S6 demonstrates a significant improvement, suggesting that the combination of DDC and LA can greatly enhance the approach’s capabilities to sense multi-scale signals and focus on disease areas. The fact that S7 achieved the highest accuracy shows that integrating different components optimizes their individual benefits, enabling the model to increase classification accuracy while maintaining low computational complexity and offering a superior solution for mobile deployment. When combined, the enhanced approach presented in this paper is capable of improving MobileNetV3 Small’s detection capacity in the task of detecting potato leaf disease, enhancing its feature extraction capabilities, and providing an effective solution for the intelligent diagnosis of potato diseases while preserving low computational complexity.

### 3.2 Model effectiveness verification

#### 3.2.1 Confusion matrix

**Figure 8:**
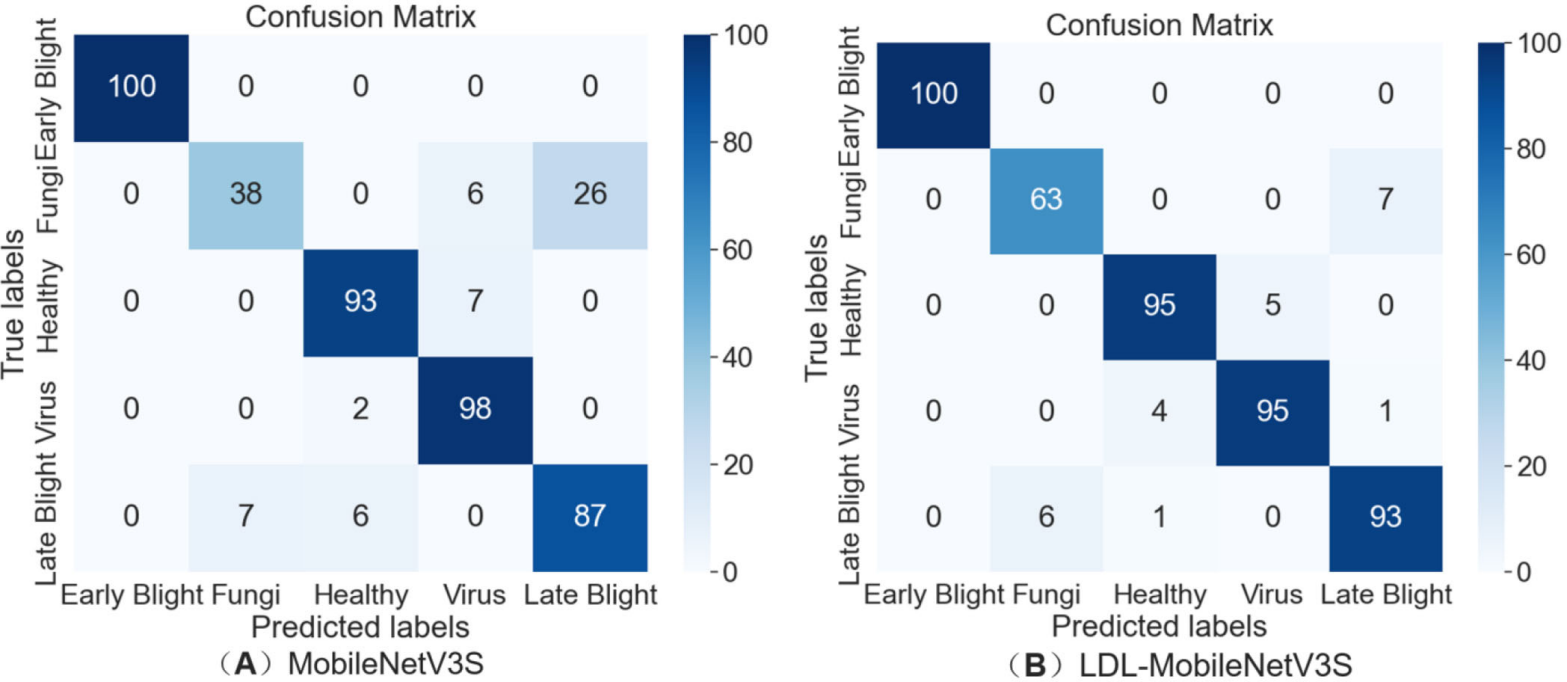
Confusion matrix for the LDL-MobileNetV3S model on the validation set. **(A)** MobileNetV3S, **(B)** LDL-MobileNetV3S.

To assess the recognition characteristics of the LDL-MobileNetV3S model for various types of potato leaf diseases, a confusion matrix diagram was created to illustrate the model’s prediction performance in a multi-category classification task, as shown in **Figure 8**. The values along the main diagonal of the confusion matrix represent the percentage of samples that were correctly classified. The model’s comprehension of the category improved with a greater value. Conversely, the off-diagonal values indicate the number of samples that were misclassified as other categories, which can reflect the degree of confusion between categories.

#### 3.2.2 Recognition performance for different disease types

To evaluate the effectiveness of the proposed LDL-MobileNetV3S model in recognizing potato leaf diseases, the trained model was tested on a designated test set. The accuracy, precision, and recall for each disease category—including Early Blight, Late Blight, Healthy, Virus, and Fungi—were calculated to comprehensively evaluate the performance of the model. The model performed satisfactorily in identifying the majority of potato leaf diseases. With an accuracy of 94.89%, it accurately categorized 446 out of the 470 test samples. **Table 6** displays the comprehensive experimental findings.

**Table 6:** Test results for different disease types.

Category	Accuracy/%	Precision/%	Recall ratio/%
Early Blight	100.00	100.00	100.00
Fungi	90.00	91.30	90.63
Healthy	95.96	96.13	96.02
Virus	95.00	95.95	95.47
Late Blight	93.94	92.08	93.17

#### 3.2.3 Heatmap visualization

**Figure 9:**
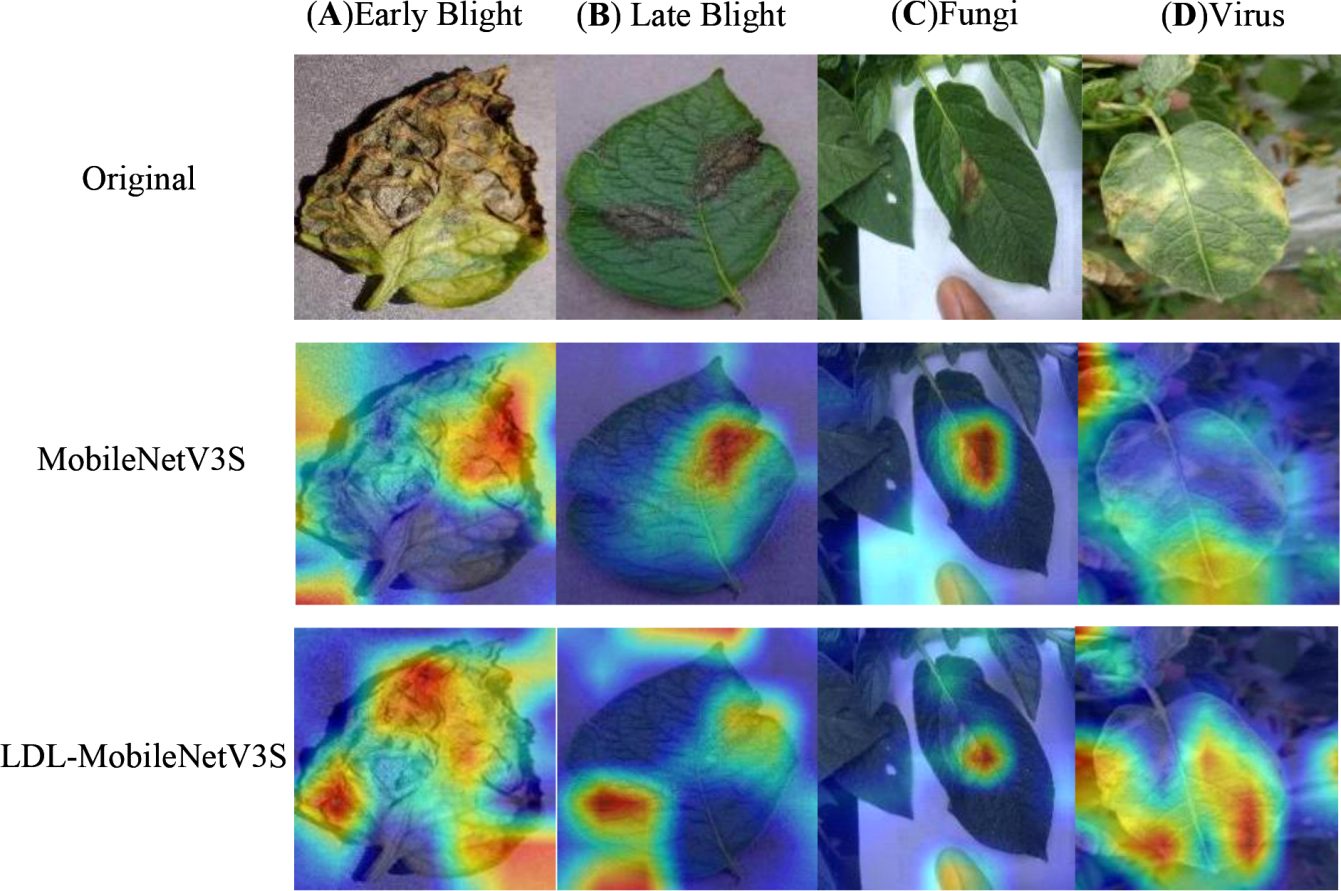
Heatmap visualization of the model. Rows from top to bottom: Original , MobileNetV3S, LDL-MobileNetV3S. Columns from left to right: **(A)** Early Blight, **(B)** Late Blight, **(C)** Fungi, **(D)** Virus.

In this study, the LDL-MobileNetV3S model is utilized to construct feature maps of disease images, and Softmax is employed to classify diseases. To examine the impact of various disease areas on the model’s classification results, which are difficult to intuitively understand based solely on the classification results, this paper employs the Grad-CAM (Gradient-weighted Class Activation Mapping) (Selvaraju et al., 2017) technique to visualize the model’s final layer of feature mapping. A selection of potato leaf disease images was chosen for the experiment, and the outcomes are seen in **Figure 9**. In the illustration, different regions are colored separately; the closer a hue is to the red area, the more strongly it correlates with the disease data.

Both the MobileNetV3 Small model and the LDL-MobileNetV3S model can focus on the disease area, as shown in **Figure 9**. However, for small and dispersed lesions such as leaf spot and early blight, the MobileNetV3 Small model is prone to losing some disease information during the feature transformation process. This results in less precise attention to critical disease locations due to its channel attention mechanism’s inability to effectively integrate spatial information to enhance feature representation; even some non-diseased areas received higher response values. On the other hand, the LDL-MobileNetV3S model employs the LF mechanism within the Bottleneck structure to improve multi-scale feature fusion. It also uses DDC to adapt to disease features at various scales, and incorporates the LA module to help the model better focus on disease areas. As a result, the model avoids misclassifying irrelevant areas and generates a more accurate response with more concentrated disease areas in the Grad-CAM heatmaps.

In summary, the LDL-MobileNetV3S model presented in this paper can greatly increase the model’s accuracy and recognition performance while more precisely concentrating on the key components of potato leaf diseases.

### 3.3 Comparison of different lightweight models

The aim of this research was to undertake a systematic comparative experiments to assess the efficacy of the proposed LDL-MobileNetV3S model in potato leaf disease detection tasks. This model was compared side by side with popular lightweight and medium complexity convolutional neural network architectures, such as ResNet18 (He et al., 2016), MobileVit (Mehta and Rastegari, 2021), MobileNetV3 (small and large versions), ShuffleNetV2 (Ma et al., 2018), ConvNeXt Tiny (Liu et al., 2022b) and EfficientNet-B0 (Tan and Le, 2019). Each model was trained using the same preprocessing procedure and training strategy on a consistent dataset. Performance metrics such as Loss, Accuracy, Precision, Recall, F1 Score, Model Size, and Parameters were evaluated on the test set. **Table 7** presents the findings from the contrasting studies.

**Table 7:** Experimental results of different comparative models.

Model	Loss	Accuracy/%	Precision/%	Recall/%	F1 score/%	Model size/MB	Params/M
Mobile Vit	0.717	77.95	78.32	77.55	76.34	5.77	1.37
ResNet18	0.468	81.58	80.84	80.87	79.89	18.34	20.30
MobileNetV3 Small	0.218	88.08	87.17	86.64	86.57	5.94	1.45
MobileNetV3 Large	0.014	89.19	89.10	88.59	88.29	16.25	4.01
ShuffleNetV2	0.247	90.05	89.04	89.18	88.28	8.97	1.20
ConvNeXt Tiny	0.343	91.72	92.02	91.72	91.77	106	27.80
EfficientNet-B0	0.023	93.23	92.86	92.04	92.20	15.61	3.83
**LDL-MobileNetV3S**	**0.020**	**94.89**	**93.54**	**92.53**	**92.77**	**6.17**	**1.50**

The bold row indicates the parameter values of the optimized LDL-MobileNetV3S model.

**Table 7** shows that classical lightweight models, including ShuffleNetV2 and MobileNetV3 Small, have previously demonstrated strong accuracy, reaching 88.08% and 90.05%, respectively. However, the accuracy of the LDL-MobileNetV3S model has increased to 94.89%, the highest among all the models studied and compared. This is in contrast to EfficientNet-B0 (93.23%) and MobileNetV3 Large (89.19%), suggesting that the three strategies—LF, DDC, and LA—suggested in the present study greatly enhance the model’s capacity to recognize complex illness characteristics.

During the model’s training and testing phases, the Loss value shows the degree of inaccuracy between the actual labels and the predicted outcomes. Compared to the original MobileNetV3 Small (0.218) and ShuffleNetV2 (0.247), the enhanced LDL-MobileNetV3S model achieves the lowest Loss on the test set, at 0.020. Even in terms of loss rate, it outperforms EfficientNet-B0 (0.023), demonstrating better generalization and convergence. This is attributed to the DDC module, which directs the model to more effectively focus on important disease regions while preventing overfitting issues.

In terms of Precision and Recall, the proposed model achieved 93.54% and 92.53%, respectively, indicating a well-balanced performance between detection accuracy and coverage. Notably, the model attained an F1 Score of 92.77%, surpassing performance-optimized models such as EfficientNet-B0 (92.20%) and ConvNeXt Tiny (91.77%), further demonstrating its superiority in comprehensive detection capability.

Depending on the model size and quantity of parameters, the study’s suggested model still maintains a high degree of lightness. While ensuring a significant increase in performance, its model size is only 6.17MB and the number of parameters is 1.509M, which is slightly higher than that of the original MobileNetV3 Small (5.94MB, 1.45M) but much smaller than that of models such as ResNet18, EfficientNet-B0 and ConvNeXt Tiny. As a consequence, this model is highly suitable for resource-constrained mobile terminals and smart detection scenarios in agricultural fields, and it has excellent adaptability for edge deployment. Notably, while maintaining about the same quantity of parameters as the first model, the improved model’s accuracy increased from 88.08% to 94.89%, and its F1 Score increased by nearly 6.2% due to structural optimization. This suggests that structural innovations are more beneficial for real-world applications than simply stacking parameters.

**Figure 10:**
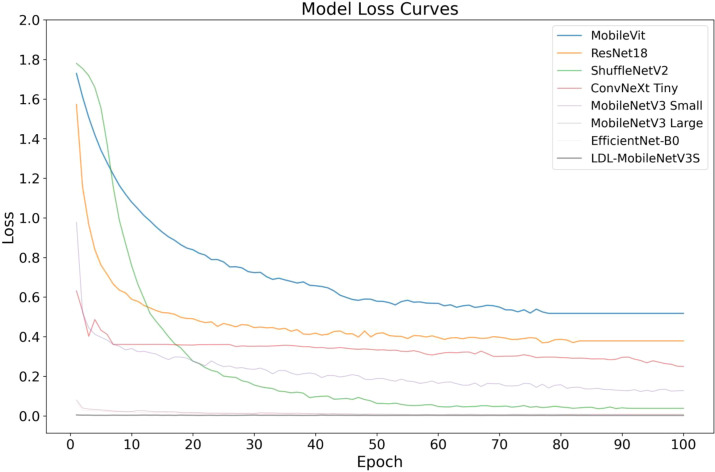
Loss curves of different models.

**Figure 11:**
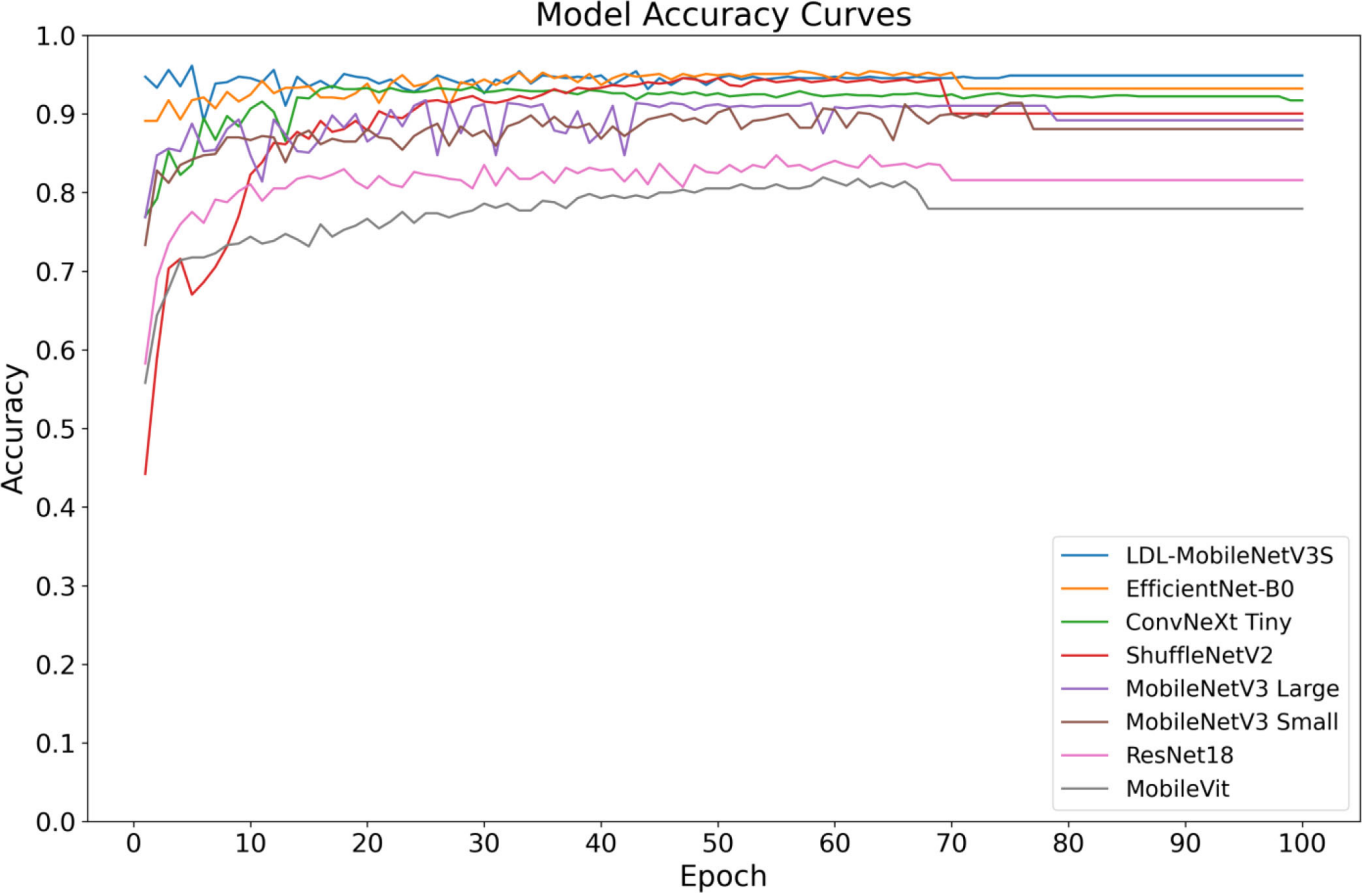
Accuracy curves of different models.

**Figures 10**, **11** shows the convergence and classification performance of each model during training. It is evident that the LDL-MobileNetV3S model can efficiently learn features from the data and converge rapidly, as shown by the low loss values during training and the rapid decline in the early stages of training. The loss curve of the current model shows more consistent and lower loss values throughout training compared to previous models (such as MobileViT and ResNet18), indicating that it is more adept at optimizing model parameters. Additionally, the model outperforms previous network architectures in terms of accuracy on the test set, demonstrating a quicker rate of accuracy improvement in the early training phases and ultimately stabilizing at approximately 94.89% at 100 epochs. In contrast, other models, including MobileViT and ResNet18, exhibit slower accuracy growth and lower final accuracy.

The high accuracy of the LDL-MobileNetV3S model is attributed to its lightweight architecture combined with the LF and DDC modules, which enable the model to more effectively capture disease features at multiple scales. In comparison, although EfficientNet-B0 and ShuffleNetV2 also demonstrate relatively strong performance, their final accuracy is lower than that of the LDL-MobileNetV3S model.

In conclusion, the enhanced model suggested in this study’s findings achieves optimal recognition performance while preserving its lightweight characteristics. In the context of crop disease detection, its strong capability in modeling multi-scale lesion regions and its effective attention-based feature selection mechanism are key contributors to the performance improvement. In contrast, although EfficientNet-B0 and ShuffleNetV2 also perform well, their final accuracy is not as high as that of the LDL-MobileNetV3S model.

## 4 Discussion

### 4.1 Impact of different improvement modules on model performance

To enhance the model’s ability to identify diverse lesion patterns in potato leaves across both controlled and field environments, the LDL-MobileNetV3S model integrates three novel modules: LF, DDC, and LA. The study examines the extent to which each module contributes to the overall performance improvement by progressively introducing the ablation experiments of the aforementioned modules, as shown in **Table 5**. This provides a quantitative basis for model structure optimization.

In particular, the LF module enhances the synergy between shallow fine-grained texture information and deep semantic information by combining feature maps at various levels. This improves the model’s ability to distinguish fine-grained lesion patterns even in complex backgrounds typical of field environments. The DDC module incorporates a learnable dilated convolution structure featuring multiple dilation rates. This structure can dynamically modify the receptive field in response to the distribution and shape of the diseased regions within the input image. This allows for the acquisition of richer information while maintaining resolution, significantly boosting the model’s robustness and generalization capabilities, and improving the recognition of diseased patches with complex shapes or fuzzy boundaries. To assist the model in concentrating on crucial regions of the image, the LA module uses a simple spatial concentration function, such as the disease region. This effectively suppresses background interference, increases the capacity of the model to perceive spatial distribution, and increases classification stability and accuracy.

Keeping the computational cost and parameter size of the model minimal, the three modules together enhance the model’s feature extraction and discrimination capabilities at various levels. In the final model (S7), which integrates all the modules, the accuracy rate reaches 94.89%, and the F1 score is 92.77%. This represents the best performance among all the experimental schemes and definitively confirms the efficacy of each structural modification.

### 4.2 Comparative analysis with mainstream lightweight models

To verify that the improved model is superior, this study conducted comparative trials using a range of representative lightweight neural network models. **Table 7** shows that while EfficientNet-B0 achieves an accuracy of 93.23%, its model size is 15.61 MB with up to 3.83 MB of parameters, which is significantly larger than the enhanced model proposed in this study. The model used in this work, however, keeps its level of complexity lower. (the model size is only 6.17 MB, and the number of parameters is only 1.50 MB), better strikes an equilibrium among efficacy and precision, and demonstrates stronger advantages in terms of lightweighting and practicality. It achieves an accuracy of 94.89% and an F1 score of 92.77%.

Furthermore, ShuffleNetV2, as a classical lightweight model, attains a 90.05% accuracy rate, which is lower than the improved model in this study across several metrics. The difference is particularly noticeable in the F1 score and recall rate, demonstrating that the structural improvements introduced in this study significantly enhance the model’s recognition ability. By improving the recognition sensitivity to multi-category disease features and optimizing overall classification performance, the model successfully reduces the rates of omission and misclassification.

In summary, based on a comprehensive evaluation of recognition accuracy, computational resource consumption, and deployment adaptability, the proposed LDL-MobileNetV3S model demonstrates superior overall performance and is more applicable to crop disease detection tasks. It provides a reliable technical foundation for intelligent disease monitoring in the context of precision agriculture.

## 5 Conclusions

In order to tackle the issues of complex spot morphology, notable scale disparities, and ineffective models in potato leaf disease image recognition, this study proposes a lightweight and effective LDL-MobileNetV3S model. The model’s sensitivity to small spots, robustness to diffuse and fuzzy spots, and responsiveness to critical areas are all enhanced by the addition of the LF, DDC, and LA modules. This creates a deep feature extraction model that better suits the requirements of agricultural applications. The model demonstrates superior performance compared to other competitive models in key metrics such as accuracy, precision, recall, and F1 score, as evidenced by its training and validation on a heterogeneous dataset comprising both laboratory and field natural photographs. It achieves a favorable balance between model compactness and high performance, maintaining a small model size and a limited number of parameters while delivering recognition performance comparable to that of larger networks.

Moreover, the model demonstrates excellent inference performance in the CPU environment. The Median Latency is 18.02 ms, indicating that most inference requests are completed within this time frame, showcasing fast real-time response capability. The 95th Percentile Latency is 22.23 ms, which demonstrates that the model’s inference process is highly stable with minimal performance fluctuations, as most inference times are below this value. The Median FPS is 55.5, meaning the model can process approximately 56 images per second, far exceeding the 30 FPS standard for real-time video streams, confirming the model’s strong real-time processing capability. These results collectively prove that the model is capable of efficient real-time inference with high throughput on resource-constrained edge devices.

The model provides a reliable and efficient approach for the identification of potato leaf disease images, holding promise for a broad spectrum of potential applications. However, the validation of its deployment in real-world application scenarios, including mobile terminals, edge computing devices, and UAV platforms, remains incomplete. While the study has demonstrated commendable performance in detecting potato leaf disease, further research is necessary to thoroughly evaluate the model’s performance metrics, specifically in terms of response time, resource utilization, and real-time performance when deployed on terminal devices. Future research will focus on expanding the field dataset to encompass multi-regional, multi-species, and multi-seasonal scenarios, as well as deploying lightweight models in real-world application contexts. Additionally, the development of a multi-task model architecture will be explored to integrate disease detection, segmentation, and severity grading into a unified framework. This work aims to facilitate the large-scale adoption of deep learning technologies in agricultural production and to provide more intelligent and precise technical support for crop disease monitoring.

## Data Availability

The original contributions presented in the study are included in the article/supplementary material. Further inquiries can be directed to the corresponding author.
